# The implementation of a GC/MS data system using DISNET: a Distributed Instrument System NETwork

**DOI:** 10.1155/S1463924684000389

**Published:** 1984

**Authors:** Paul J. Gemperline, Robert Megargle

**Affiliations:** 1 Department of Chemistry Cleveland State University Cleveland Ohio 44115 USA; 2 Department of Chemistry East Carolina University Greenville North Carolina 27834 USA


Journal of Automatic Chemistry, Volume 6, Number 4 (October–December 1984), pages 197–201

### The implementation of a GC/MS data system using DISNET: a Distributed Instrument System NETwork

**Paul J. Gemperline and Robert Megargle**

*Department of Chemistry, Cleveland State University, Cleveland, Ohio 44115, USA*

### Introduction

The primary goal of a computer network is to achieve resource sharing between several computers. Resource sharing is most easily performed at the application program level, with lower layers of software providing communications services. Application programs which make local data, peripherals, or processes available for shared use are defined as 'resource providers'. Application programs which require such resources from the network are defined as 'resource consumers'. This distinction helps the system designer identify the qualities of resource providers and consumers necessary to address data communication problems in a straightforward manner, while ensuring the effort invested will provide satisfactory solutions. We have developed this resource sharing protocol for the Distributed Instrument System NETwork (DISNET) to provide an environment suitable for the development and growth of a distributed system for real-time instrument control, data acquisition, and computation in a laboratory environment.

### Resource sharing protocol

Several desirable qualities of resource providers were identified during the design phase of this project. First, a resource provider should always be ready and waiting for use by any remote station. If the host computer of the resource is capable of performing other tasks, the resource provider software should not present a significant load to the host computer system while it is waiting to be called. Secondly, the resource provider should make a short set of commands available to the resource consumer. This process should allow the consumer to send a packet of data containing the command to be performed, and, in most cases, the data which is to be manipulated by the command. Third, resource providers which serve multiple consumers must keep data packets from independent consumers separate and distinct. Any errors in the transactions from one consumer must not affect the others. Finally, after sending command packets, the consumer should wait for the provider to send an acknowledgement packet and any associated results. The acknowledgement packet informs the consumer when a function is complete or if any errors occurred. This scheme ensures that communicating tasks remain synchronized. The resource consumer is prevented from sending multiple sets of commands and data faster than the provider can process them. In return, the consumer is guaranteed to be informed about any failures which may have occurred during the processing of a command before any additional activity can proceed.

### Resource sharing in the DISNET environment

The DISNET system, with its channels, supervisor calls, and program scheduling capabilities [1 and 2], allows the programmer to efficiently implement the resource consumer/provider protocol. The 'enable channel' call allows a resource provider to make itself available for use. Once a channel is enabled, the provider can suspend itself and lie dormant until a consumer in a remote station performs an 'open channel' call, causing an active channel link to be created. The DISNET Channel Control Module (CCM) dynamically creates these active channel links for application programs and prevents third parties from interfering. Providers can enable multiple channels so that many consumers can gain assess to a common resource. The CCM guarantees that data from multiple channels remains separate, preventing multiple consumers from interfering with each other. In this case, the provider must poll its channels in response to channel events to determine which consumer requires service. The 'DONE' flag and return codes in the channel control block (CCB) provide the necessary channel status and information.

Once a channel has been opened, the 'receive data' call allows the provider to wait for command information from the consumer. The provider next suspends itself by performing a 'wait' call. The provider is immediately rescheduled when the command information is received from a channel. Several channels could be waiting for data in such a fashion. Each time the resource provider is rescheduled it polls the individual CCBs to determine which consumer requires service.

The resource consumer initiates the performance of all functions by opening a channel to the resource provider and sending command packets and data. Since the 'open' and 'send' calls proceed to completion, the consumer need not suspend itself or poll CCBs to determine when these functions have been completed. All that need be checked is the return code.

Once a command packet has been sent, the consumer must perform a 'receive data' call and then issue a 'wait' call to suspend itself until the data is received. There is always a possibility that the provider may fail and never return the acknowledgement packet. For this reason, the 'wait' call at the consumer must have a time-out delay long enough for the provider to complete the function and return the acknowledgement. The consumer is rescheduled immediately when the acknowledgement packet is received and any remaining 'wait time' cancelled. The successful

Paul Gemperline is now based at the Department of Chemistry, East Carolina University, Greenville, North Carolina 27834, USA. Correspondence should be addressed to Robert Megargle.

completion of the operation is indicated by the appropriate combination of the 'DONE' flag, return code, and the data in the acknowledgement packet. If the acknowledgement packet is never received, the consumer will eventually be rescheduled at the end of its time-out delay. By testing the 'DONE' flag in the CCB, the consumer will know that the provider failed to perform its function in the required time interval.

Resource consumers can open channels to several providers at once. The CCM automatically routes command information and data to the proper resource, whether the resources reside in the same host or are widely dispersed throughout the network. Finally, the 'close channel' command allows either the consumer or provider to close access to the resource provider. At this point the resource provider can enable another channel and wait for its service to again be requested.

### Prototype resource provider

A prototype disk resource provider has been written for a Texas Instruments Model 960A minicomputer and Diablo Model 33 disk drive. This resource supports commands to open, close, create, or delete random access disk files. An input/output command is provided to read or write the files. A new disk file system was written since the original operating system supplied by Texas Instruments did not support the necessary functions. The new file system uses a managed area on disk which grows toward the end of the disk as files are added. Approximately 20% of the disk space is reserved to maintain compatibility with the original operating system. The remaining 80% is available for use by the new file system. All files maintained by the new system are accessed by 12-character file names. Blank disk space is automatically reclaimed when files are deleted from the middle of the managed area.

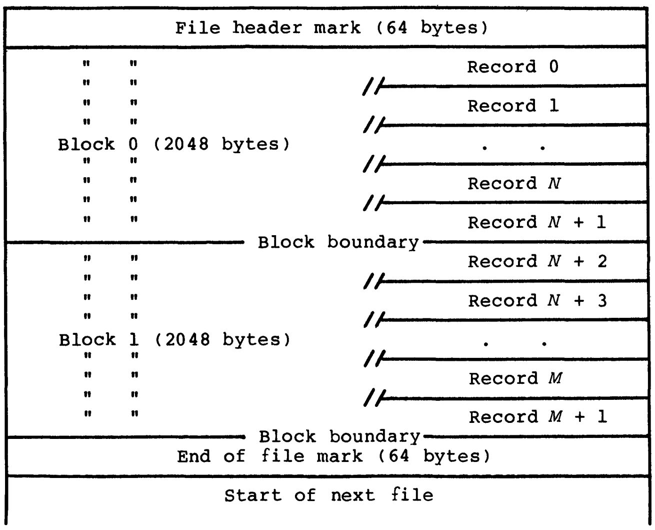

*Figure 1. Random access file format.*

Figure 1 shows the format of individual files. A file header is used to mark the beginning of each file and contains the file name, file length, and date and time of creation. Files are accessed from the disk in blocks of 2048 bytes regardless of data record size to reduce the number of disk accesses, thereby improving throughput and response time. When data records are smaller than 2048 bytes, several records are packed into the block to make efficient use of disk space. Records span across block boundaries when the block size is not evenly divisible by the record size, yielding even greater economies in disk space.

Block spanned records also permit record sizes greater than 2048 bytes. The system supports random access by calculating the location of individual data records within files. Records are extracted from blocks and returned to application programs for read operations and updated for write operations. The number of block write operations for updated blocks are minimized by deferring them until new blocks are needed.

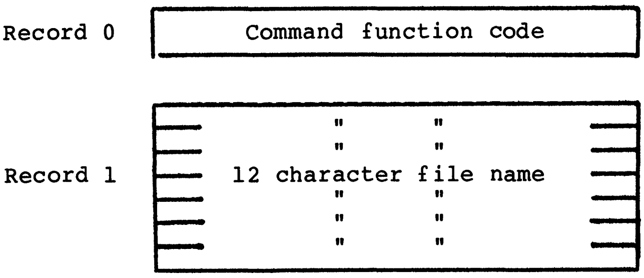

*Figure 2. Disk resource command format.*

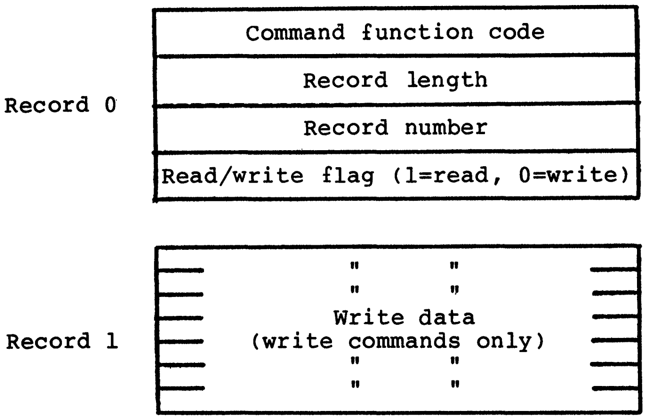

*Figure 3. Format of read/write command.*

The prototype disk resource provider uses the above file system to make five general-purpose commands available. Two other special-purpose commands are described later. The five basic commands allow resource consumers to create, delete, open, and close disk files referenced by 12-character name, and perform read/write operations to open files. The consumer is required to send two packet records [2] for the create, delete, open and close commands (see figure 2). The first packet record contains two bytes and specifies the function to be performed. The second packet record specifies a 12 byte file name. The format of the read/write command is shown in figure 3. The first packet record specifies the command code, the length of a record in the disk file, the disk file record number, and a read/write flag. Write commands require a second packet record containing the write data. The read/write command can only be used on open channels.

An acknowledgement transaction, consisting of one or two packet records, is transmitted to verify the successful or unsuccessful completion of these five operations. Its format is shown in figure 4. The first packet record is always transmitted and contains an acknowledgement flag. The second packet record is only transmitted for the read function and contains the data read by the function.

A special command is provided to obtain a map of the managed area on the disk. The format of the command is shown in figure 5. The START/CONTINUE flag instructs the resource provider to initialize the mapping operation or continue with the current one. One acknowledgement packet containing two packet records is returned each time the command is performed

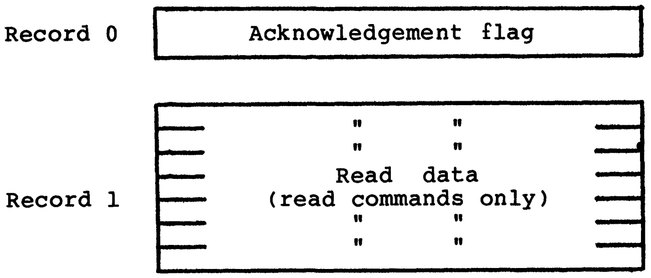

*Figure 4. Format of acknowledgement transaction.*

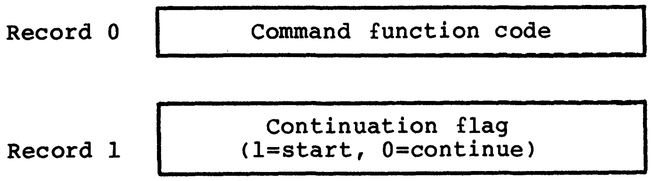

*Figure 5. Disk map command format.*

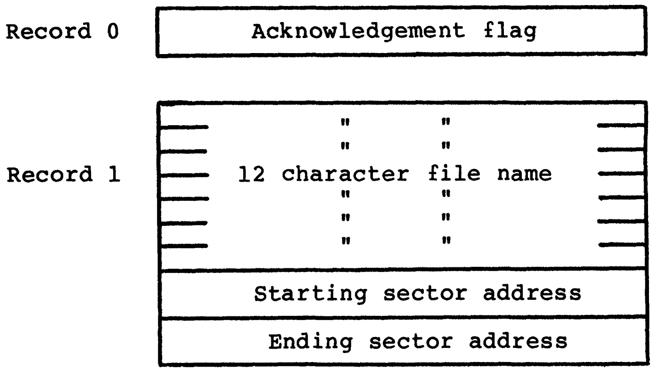

*Figure 6. Disk map acknowledgement transaction.*

(see figure 6). The first packet record contains the acknowledgement flag and the second packet record contains a 12 byte file name, plus starting and ending disk addresses. One file name is returned to the consumer each time the command is performed and a file name of binary zeros is returned when the end of files is reached.

### GC/MS data systems

A custom GC/MS data system has been developed using the prototype file system. The data system controls a Finnigan model 3200 GC/MS. A Tektronics storage oscilloscope and a Zeta digital X-Y plotter provide for high-speed graphics. A Texas Instruments Silent 700 terminal with twin cassette-tape drives is used for operator communication and local mass storage. The computer-to-GC/MS interface was built at Cleveland State University and consists of a 12 bit integrating analogue-to-digital converter for measuring mass intensities and a 16 bit digital-to-analogue converter to focus the mass spectrometer to a desired mass-to-charge ratio.

The software for the data system provides all of the usual features found on commercial quadrupole data systems, including repetitively scanned data acquisition, data display, and a unique quantitative analysis system. All of the functions can use DISNET in some phase of their operation. Cassette-tapes can be used in the event of DISNET failures, although at greatly reduced speed and capacity. The lack of fast, random access, mass storage on the GC/MS computer system provided the impetus for developing the DISNET file resource.

The prototype file system runs continuously at one network node and is available for immediate use by the GC/MS node. Single line commands at the GC/MS terminal allow the GC/MS operator to obtain a list of all remote files; create, read, or delete mass spectral files; or download object programs. In each case, the files are accessed by name. The disk resource provider node stores download object files as sequential 80 byte card images, one card per record. It saves the mass spectral files with one mass spectrum per record. The GC/MS data acquisition and data display routines use the remote disk resource by default and prompt the operator for file names. The usual display functions, such as background subtraction and summation of spectra, are provided for post-processing data.

Two disk files are created during a GC/MS run; a Reconstructed Gas Chromatogram file (RGC) and a data file. The RGC file contains the run-time parameters and the summed intensities of each mass spectrum to simulate gas chromatography data. Each record in the data file contains an individual mass spectrum. A header for each spectrum contains additional run-time parameters and sufficient information to calculate the record length of individual mass spectra in the data file. The random-access mechanism supported by the resource provider makes retrieval of individual spectra trivial.

Several benefits were immediately realized once the DISNET disk resource became operational. The time required to load programs was greatly reduced. For example the data-acquisition program required over 10 min to load from cassette tape. The load time has been reduced to less than 8 s with the DISNET disk resource. High scan speeds are easily achieved when the DISNET disk resource is used for data storage. Data acquisition rates of nearly 1000 16 bit points/s are now possible. A double buffering scheme for acquired data works so well that there is no noticable dead time between scans. Using cassette-tape, data-acquisition rates no greater than 150 points/s were possible because of the slow recording rate. Significant savings in time have also been realized when the DISNET disk resource is used to retrieve mass spectral data. From a file of over 500 spectra, any single spectrum can be retrieved in less than 0·5 s. Up to 20 min were required to retrieve a single spectrum when cassette-tape was used and the desired spectrum was near the end of the tape.

### Distributed processing

The addition of fast random-access mass storage has made it possible to add new functionality to the GC/MS data system, which would have been prohibitively time-consuming using cassette-tape. One example is the addition of the 'accumulate mass chromatogram' command to the disk resource for post-processing mass spectral data. This command causes the disk resource to collect the intensity data for a single ion from a specified data file. The results are returned to the GC/MS data system in the RGC file format for processing by the same powerful graphics software used to display an RGC.

The 'accumulate mass chromatogram' is a unique example of distributed processing. The remote file system calculates chromatographic profiles locally and sends only the results to the GC/MS data system. This approach greatly reduces the communication overhead that would have been required if each mass spectrum were to be sent to the GC/MS system for the intensity at the appropriate m/e to be read. As a result, much less time is required to process the command. Future enhancements, such as real-time spectral searching, could be programmed in the remote disk resource without affecting data-acquisition rates in the GC/MS data system.

### GC/MS data system test

The DISNET version of the GC/MS data system has been extensively tested and routine use of it began in 1982. Typical results are presented here to demonstrate the capabilities of the general-purpose data-acquisition, data display, and mass chromatogram software. A sample of polychlorinated biphenyls (PCB) (Aroclor 1254 in isooctane, 1·0 microgram/microlitre, Supelco, Inc.) was chosen because of the complexity of the mixture and the extensive amount of research already done on these materials. The mixture is 54% chlorine by weight and contains measurable amounts of monochloro- through hepta-chlorobiphenyl compounds, with hexachlorobiphenyl isomers predominating [3]. Over 210 different compounds are theoretically possible and most geometric isomers within groups (di-substituted, tri- substituted etc.) cannot be resolved by the low resolution packed column used in these experiments. The complexity of the mixture is an advantage, however, in demonstrating the mass chromatogram display functions. Several co-eluting chromatographic peaks are clearly deconvoluted by the software.

The data was obtained using a 1·5 m × 2 mm Pyrex 'U' column, packed with 3% OV-101 on 80/100 mesh chromosorb from Alltech, Inc. Injection volumes of one microlitre were used and the column temperature programmed from 190°C to 280°C at 4°C/min. Methane (Matheson Gas Products, 99·95%) was used as the carrier gas. The GC effluent was admitted directly into the chemical ionization source where the methane functioned as the reagent gas. The ion source pressure was maintained at 650 microns, the source temperature was maintained at 75°C, and an ionization energy of 150 eV was used. Figure 7 shows the reconstructed chromatogram of the mixture. All of the methane chemical ionization (CI) spectra of the PCBs are characterized by strong $M+1^+$ peaks, less intense peaks at $M+29^+$ and weaker $M+41^+$ peaks. Clusters, which are characteristic of chlorine-containing compounds, are observed due to the naturally occurring isotopes of chlorine; $Cl^{35}$ and $Cl^{37}$. Spectra uncorrected for background are shown in figures 8 and 9 and correspond to the chromatographic peaks labelled numbers 10 and 11 respectively in figure 7. The cluster at m/e 291 to 297 in figure 8 are $M+1^+$ type peaks from tetrachlorinated biphenyl isomers. Two additional clusters of peaks are observed from m/e 319 to 325, corresponding to $M+29^+$ type peaks, and from m/e 331 to 337, corresponding to $M+41^+$ type peaks. Interfering peaks are observed from m/e 325 to 333, m/e 353 to 361, and m/e 365 to 371.

Pentachlorinated isomers would be expected to show clusters from m/e 325 to 333, m/e 353 to 361, and m/e 365 to 373, corresponding to $M+1^+$, $M+29^+$, and $M+41^+$ type peaks. The interfering peaks in figure 8 can be accounted for if a small

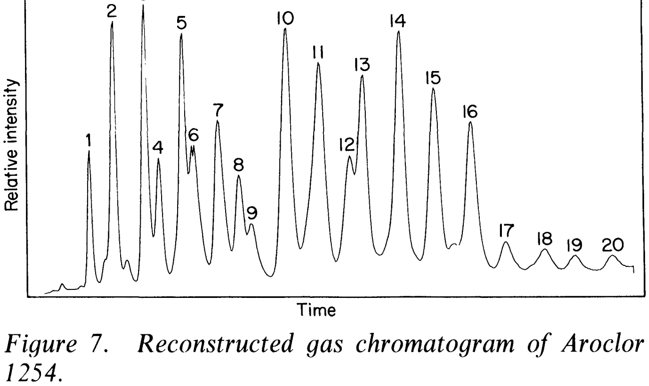

*Figure 7. Reconstructed gas chromatogram of Aroclor 1254.*

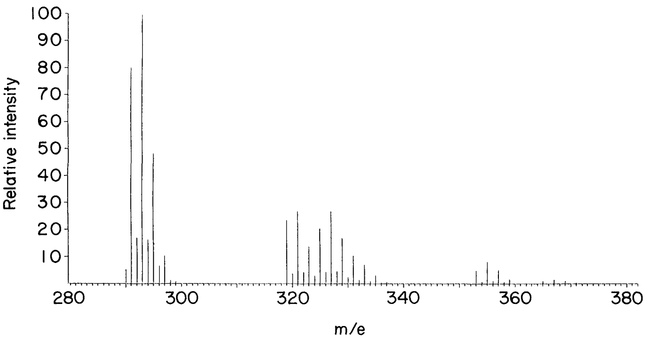

*Figure 8. Mass spectrum of Peak 10.*

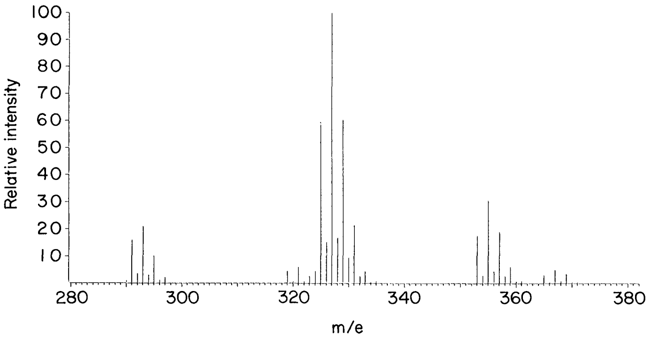

*Figure 9. Mass spectrum of Peak 11.*

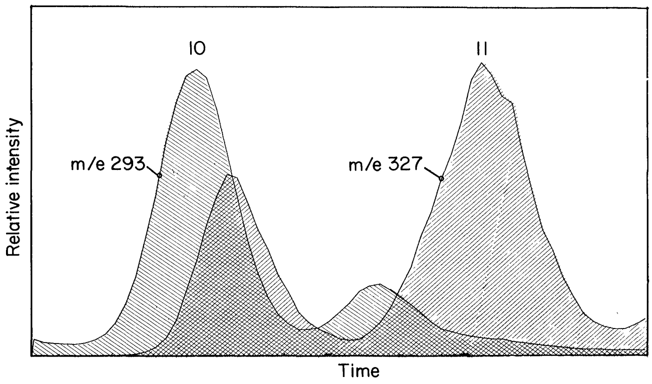

*Figure 10. Mass chromatogram of Peaks 10 and 11.*

amount of the pentachlorinated isomers co-elute with the tetrachlorinated compounds. The spectrum in figure 8 indicates that the tetrachlorinated isomers predominate. The spectrum in figure 9 also shows peak clusters which can be accounted for by the isomers of both tetra- and pentachlorinated biphenyls. The reverse situation is observed, however, in that the pentachlorinated isomers predominate.

Two conditions could account for the interferences observed in these two spectra. First, excessive peak tailing could lead to cross-contamination. Alternatively, sufficient chromatographic resolution may be available to separate geometric isomers of both the tetra- and penta-substituted biphenyls into two or more peaks, each containing chromatographically similar isomers. Co-elution of these peaks could give rise to the interfering mass fragments observed in the spectra.

The mass chromatogram display routine has been used to verify the later case. Figure 10 shows a plot of the ion current for only the m/e 293 and m/e 327 fragments. The ion current for m/e 293 is proportional to the concentration of the tetrachlorinated biphenyls and the ion current observed at m/e 327 is proportional to the concentration of the pentachlorinated biphenyls with a slight contribution from the tetrachlorinated biphenyls. Four well resolved peaks are seen to be present. Peaks 10 and 11 are each composed of at least two co-eluting peaks as shown by the mass chromatogram in figure 10.

### Conclusions

Distributed processing offers significant advantages in scientific laboratories. Various resource providers, like disk files, printers, and plotters, can be shared for greater economy. With a variety of resource providers in place, individual instrument systems or experimental set-ups can be designed more easily and implemented with lower cost in both time and money. Major subsections of such systems are already present and available to new endeavours from the nearest network port. There are also interesting possibilities to be explored with regard to sharing and combining data from different instrument or computational sites. Imaginative approaches now become possible to improve the knowledge and information gained from laboratory experiments.

### References

1. Gemperline, P., Megargle, R., Dartt, A., Slivon, L. and Zadnik, V., *Journal of Automatic Chemistry*, **6** (1984), 27.

2. Gemperline, P. and Megargle, R., *Journal of Automatic Chemistry*, **6** (1984).

3. Sissons, D. and Welti, D., *Journal of Chromatography*, **60** (1961), 15.

### READER ENQUIRY SERVICE

Each advertisement and each editorial item in the Product News section in *Journal of Automatic Chemistry* carries a number which corresponds with a number on the Reader Enquiry Card bound into every issue.

If there are any items on which you would like further information, circle the relevant number on the card, indicate the volume and issue number, fill in your name and address, and return the card to the Marketing Department, Taylor & Francis Ltd, Rankine Road, Basingstoke, Hampshire RG24 0PR, UK. Your card will then be forwarded to the company/ies concerned for action.

*Through using the Reader Enquiry Service, you can obtain information on a variety of products through one simple operation.*

### BOOKS

The Editor is anxious to establish a book review section in *Journal of Automatic Chemistry*. He would welcome readers who are about to publish a book, or having a paper in a proceedings volume, either asking their publisher to send a review copy to him or letting him know about the publication.

### NOTES FOR AUTHORS

*Journal of Automatic Chemistry* covers all aspects of automation and mechanization in analytical, clinical and industrial environments. The *Journal* publishes original research papers; short communications on innovations, techniques and instrumentation, or current research in progress; reports on recent commercial developments; and meeting reports, book reviews and information on forthcoming events. All research papers are refereed.

### Manuscripts

Two copies of articles should be submitted to the Editor. All articles should be typed in double spacing with ample margins, on one side of the paper only. The following items should be sent: (1) a title-page including a brief and informative title, avoiding the word 'new' and its synonyms; a full list of authors with their affiliations and full addresses; (2) an abstract of about 250 words—this should succinctly describe the scope of the contribution and highlight significant findings or innovations; it should be written in a style which can easily be translated into French and German; (3) the main text with sections and subsections numbered; (4) appendices (if any); (5) references; (6) tables, each table on a separate sheet and accompanied by a caption; (7) illustrations (diagrams, drawings and photographs) numbered in a single sequence from 1 upwards and with the author's name on the back of every illustration; captions to illustrations should be typed on a separate sheet. Papers are accepted for publication on condition that they have been submitted only to *Journal of Automatic Chemistry*.

### References

References should be indicated in the text by numbers following the author's name, i.e. Skeggs [6]. In the reference section they should be arranged thus:

to a journal

Manka, D. P., *Journal of Automatic Chemistry*, **3** (1981), 119.

to a book

Malmstadt, H. V., in *Topics in Automatic Chemistry*, Ed. Stockwell, P. B. and Foreman, J. K. (Horwood, Chichester, 1978), p. 68.

### Illustrations

Line diagrams are preferred to photographs. Original copies of diagrams and drawings should be supplied, and should be drawn to be suitable for reduction to the page or column width of the *Journal*, i.e. to 85 mm or 179 mm, with special attention to lettering size. Photographs may be sent as glossy prints or as negatives.

### Proofs and offprints

The principal or corresponding author will be sent galley proofs for checking and will receive 50 offprints free of charge. Additional offprints may be ordered on a form which accompanies the proofs.

*Manuscripts should be sent to the Editor: Dr Peter B. Stockwell, P.S. Analytical Ltd, 2 Eagles Drive, Tatsfield, Westerham, Kent TN16 2PB, UK.*

